# Ulcerative colitis relapse after *Helicobacter pylori* eradication in a 12-year-old boy with duodenal ulcer

**DOI:** 10.1186/s12876-021-02010-1

**Published:** 2021-11-10

**Authors:** Yuji Fujita, Keiichi Tominaga, Takanao Tanaka, Takeshi Sugaya, Shigemi Yoshihara

**Affiliations:** 1 Department of Pediatrics, Dokkyo Medical University, 880 Kitakobayashi, Mibu, Shimotsuga, Tochigi 321-0293 Japan; 2 Department of Gastroenterology, Dokkyo Medical University, Tochigi, Japan

**Keywords:** Fecal calprotectin, *Helicobacter pylori*, Ulcerative colitis

## Abstract

**Background:**

*Helicobacter pylori* (*H. pylori)* prevalence is lower in patients with inflammatory bowel disease (IBD) than in those without IBD, suggesting that *H. pylori* plays a protective role in IBD. It has been reported that IBD may occur due to *H. pylori* eradication; however, it is unclear whether *H. pylori* eradication increases the incidence of IBD. Moreover, the effect of *H. pylori* eradication on IBD activity is unclear.

**Case presentation:**

An 11-year-old boy diagnosed with ulcerative colitis (UC) was in clinical remission, with treatment involving 5-aminosalicylic acid. Fecal calprotectin (FC) level had decreased to 33.2 mg/kg, indicating mucosal healing. At age 12, he experienced epigastric pain on an empty stomach, which was relieved with dietary intake. His FC level was elevated without UC symptoms, such as diarrhea and bloody stools. He was diagnosed with *H. pylori* duodenal ulcer. *H. pylori* eradication (clarithromycin and amoxicillin for 7 days and a proton-pump inhibitor) led to symptom improvement the day after treatment initiation. However, he developed diarrhea and his FC level remained high despite improvement in duodenal ulcer symptoms and endoscopic findings of *H. pylori* eradication. Colonoscopy results indicated UC relapse.

**Conclusions:**

*H. pylori* eradication may worsen UC activity. However, further studies are required as this case report involved only one pediatric patient with increased UC activity after *H. pylori* eradication*.*

## Background

The prevalence of inflammatory bowel disease (IBD), including ulcerative colitis (UC) and Crohn’s disease, has increased worldwide. [1] Its pathogenesis involves gut microbiota, [2] including *Helicobacter pylori* (*H. pylori*). The prevalence of *H. pylori* is lower in patients with IBD than in those without IBD, which suggests *H. pylori* plays a protective role in the development of IBD. [3, 4] It has previously been reported that IBD may occur due to *H. pylori* eradication [5–7]; however, whether *H. pylori* eradication increases the incidence of IBD as well as the effect of *H. pylori* eradication on IBD activity remains unclear. We describe a pediatric case of UC, worsened through *H. pylori* eradication for the treatment of duodenal ulcer.

### Case presentation

**Fig. 1:**
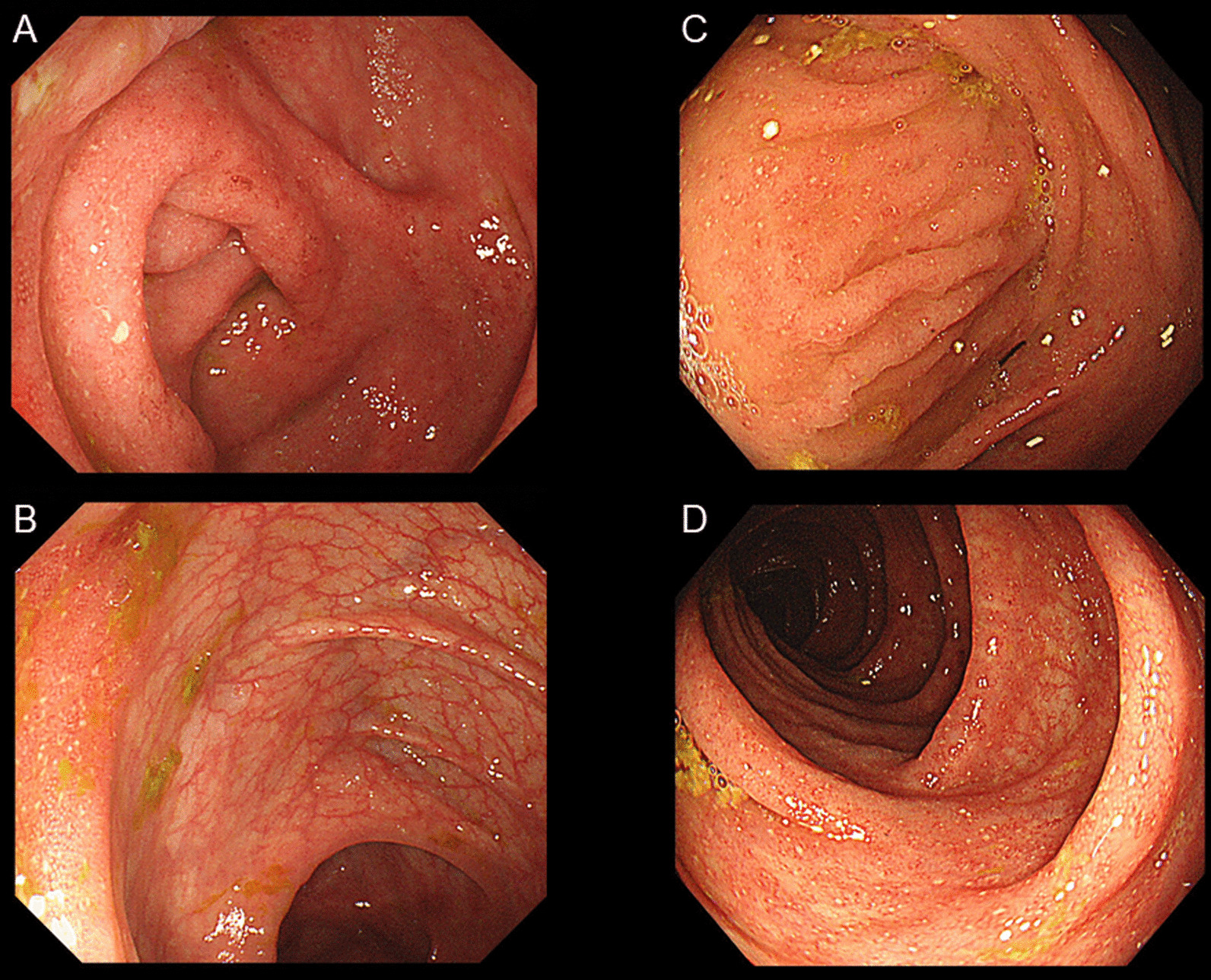
Colonoscopy findings. The cecum (**a**) and ascending colon (**b**) at the time of diagnosing ulcerative colitis with slight inflammation from the rectum to the transverse colon. The cecum and ascending colon show loss of vascular permeability and adherent purulent mucus. At the time of ulcerative colitis relapse, the cecum (**c**) and ascending colon (**d**) had small aphthae and edematous mucosa

**Fig. 2:**
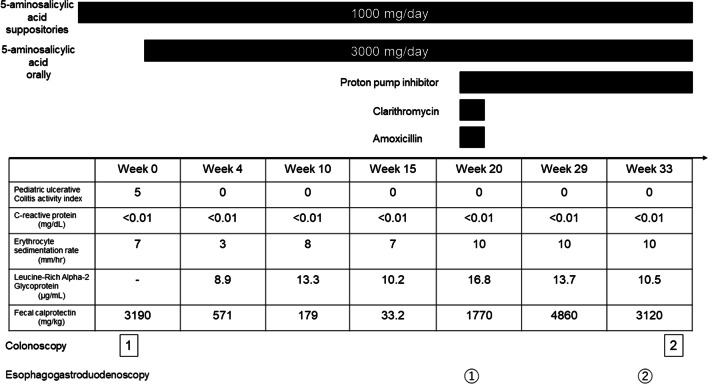
A timeline showing the patient’s disease course in terms of pediatric ulcerative colitis activity index values, C-reactive protein, erythrocyte sedimentation rate, leucine-rich alpha-2 glycoprotein, and fecal calprotectin levels at different time points. The timeline and duration of various treatments administered for ulcerative colitis and *Helicobacter pylori* are indicated*.* The time points indicating when the colonoscopy and the esophagogastroduodenoscopy were undertaken are also shown

An 11-year-old boy consulted a family doctor for bloody diarrhea. The patient had no specific medical or family history. A colonoscopy from the rectum to the sigmoid colon led to the diagnosis of pediatric UC with a pediatric ulcerative colitis activity index (PUCAI) of 30. He was administered 5-aminosalicylic acid (5-ASA) suppositories (1 g/day) and was referred to our hospital for subsequent treatment. His symptoms promptly improved to a PUCAI of 5. His blood examination results were unremarkable but the fecal calprotectin (FC) level was elevated to 3,190 mg/kg. A complete colonoscopy was performed, which revealed inflammatory findings from the rectum to the transverse colon. Moreover, the cecum and ascending colon showed loss of vascular permeability and adherent purulent mucus (Fig. 1a, b). The patient was prescribed oral 5-ASA (3,000 mg/day). The FC level gradually decreased to 33.2 mg/kg by week 15 (Fig. 2).

**Fig. 3:**
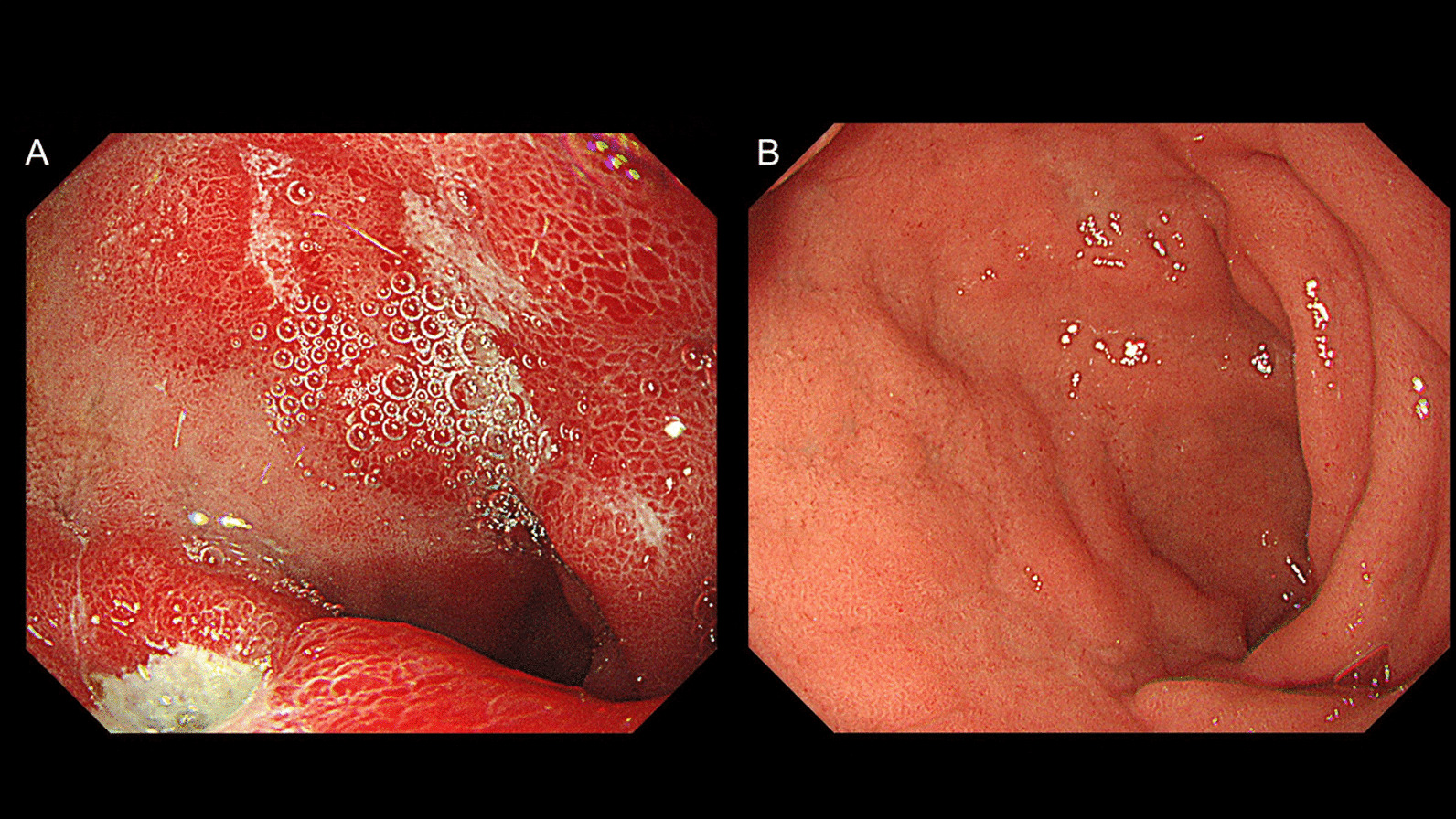
Esophagogastroduodenoscopy. **a** At the time of duodenal ulcer diagnosis, esophagogastroduodenoscopy findings revealed an A1 ulcer on the lower wall of the duodenal bulb. **b** Three months after *Helicobacter pylori* treatment, esophagogastroduodenoscopy findings indicated healing of the duodenal ulcer and scarring

At 12 years old, he complained of epigastric pain on an empty stomach, which was relieved with dietary intake (week 19). He reported no UC symptoms, including diarrhea and bloody stools (PUCAI, 0); however, an elevated FC level was noted (week 20). Esophagogastroduodenoscopy (EGD) findings indicated an A1 ulcer on the lower wall of the duodenal bulb (Fig. 3A). A rapid urease test was positive, and he was diagnosed with a duodenal ulcer due to *H. pylori* infection. The *H. pylori* infection was treated using clarithromycin and amoxicillin for 7 days and a proton-pump inhibitor. The patient’s symptoms improved the day after treatment initiation. In week 33, an EGD was performed to evaluate the therapeutic effect. The duodenal ulcer had healed, and scarring was observed (Fig. 3B).

The FC level remained high despite improvement of duodenal ulcer symptoms and endoscopic findings of *H. pylori* eradication. The patient subsequently developed diarrhea (PUCAI, 10) in week 35 and a UC relapse was considered. Cytomegalovirus antibody, antigenemia, and tuberculosis (T-SPOT assay) tests were negative. Colonoscopy showed small aphthae and edematous mucosa throughout the colon in week 36 (Fig. 1C, D). Histopathological examination revealed UC lesions grades 3–5 based on the Matts classification. The patient was diagnosed with a UC relapse, and the 5-ASA dosage was increased to 4,000 mg/day (100 mg/kg/day).

## Discussion and conclusion

*H. pylori* has been considered protective against immune-mediated diseases, including IBD. However, it has additionally been associated with peptic ulcers and gastric cancer. *H. pylori* eradication may be more effective in preventing the development of gastric cancer in people of Asian ethnicity than in those of European ethnicity; therefore, most patients of Asian ethnicity actively undergo *H. pylori* treatment. [8] In Asia, the incidence of IBD has been increasing, [9] and theoretically, active eradication of *H. pylori* may have a role in this observed increase. Several studies have reported the development of IBD after eradication of *H. pylori*. [5–7] Furthermore, there is a report that there is an inverse association between *H. pylori* and UC severity. [10] However, data concerning the effect of *H. pylori* eradication on IBD activity are limited. A multicenter retrospective cohort study showed that *H. pylori* eradication did not worsen IBD activity. [11] However, there have been no reports involving pediatric patients with IBD. We report a pediatric patient with worsened UC activity following the eradication of *H. pylori.*

The mechanism by which *H. pylori* acts protectively on IBD is unclear; however, there are various reports using mouse models. Zhang et al. reported that *H. pylori* had a protective action in mice with chronic experimental colitis. They considered that this protective mechanism involved *H. pylori* colonization increasing regulatory T cells and interleukin (IL)-10 and suppressing IL-17 producing effector T-helper cells. [12] Gravina et al. reported that Hp(2–20) derived from H. pylori accelerates the healing of not only gastric mucosa but also inflamed colonic mucosa in 2-, 4-, 6-trinitrobenzenesulfonic acid-induced colitis, and the effect is considered to be associated with the reduction of inflammatory mediators such as tumor necrosis factor-α in colonic tissue. [13] Chen et al. reported that NLRP12 decreased MCP-1 and MIP-1α expression in intestinal epithelial cells from the analysis of the exomes derived from *H. pylori*-infected IBD patients, and this report showed NLRP12 has a negative correlation with the disease activity in pediatric IBD patients. [14] Further research on the function of *H. pylori* in IBD patients is required.

In our patient, endoscopic mucosal healing was not confirmed after the initial 5-ASA treatment. However, an FC level < 250 mg/kg is indicative of endoscopic and histological mucosal healing. [15] Based on these findings, we considered that our patient had achieved remission after the initial treatment. We noted elevation in his FC level following the onset of the duodenal ulcer; however, it was unclear whether this elevated FC level due to the duodenal ulcer or to UC relapse. However, there were no clinical symptoms of UC. Three months after *H. pylori* eradication, the patient had an increased FC level, and he gradually developed diarrhea. Therefore, the elevated FC level was considered to be due to UC activity. The FC level is the most helpful biomarker of endoscopic disease activity. [16] No other hematological biomarkers, including leucine-rich alpha 2 glycoprotein, are considered helpful in evaluating IBD activity. [17] FC monitoring following *H. pylori* eradication may help to detect endoscopic and histological relapse prior to clinical relapse. Endoscopic and histological remission in addition to clinical remission should be confirmed in patients with *H. pylori-*induced peptic ulcer cases.^18^.

To date, no studies concerning pediatric IBD have evaluated the FC level prior to and post *H. pylori* eradication. Measuring the FC level is non-invasive and allows early detection of relapse during the asymptomatic phase. Further studies are required to determine the effect of *H. pylori* eradication in relation to pediatric IBD activity.

## Data Availability

Data sharing is not applicable to this article as no datasets were generated or analyzed during the current study.

## Abbreviations

EGD: Esophagogastroduodenoscopy
FC: Fecal calprotectin
*H.**pylori*: Helicobacter pylori
IBD: Inflammatory bowel disease
IL: Interleukin
PUCAI: Pediatric Ulcerative Colitis Activity Index
UC: Ulcerative colitis
